# Transcriptomic profiling of long non-coding RNAs and messenger RNAs in the liver of mice during *Toxoplasma gondii* infection

**DOI:** 10.1186/s13071-023-06053-z

**Published:** 2024-01-16

**Authors:** Yang Zou, Xing Yang, Chao Chen, He Ma, Hong-Wei Cao, Jing Jiang, Xin-Yu Wei, Xiao-Xuan Zhang

**Affiliations:** 1 School of Pharmacy, Yancheng Teachers University, Yancheng, Jiangsu Province 224002 People’s Republic of China; 2 School of Life Sciences, Baicheng Normal University, Baicheng, Jilin Province, 137000 People’s Republic of China; 3 State Key Laboratory of Veterinary Etiological Biology, Key Laboratory of Veterinary Parasitology of Gansu Province, Lanzhou Veterinary Research Institute, Chinese Academy of Agricultural Sciences, Lanzhou, Gansu Province 730046 People’s Republic of China; 4 Department of Medical Microbiology and Immunology, School of Basic Medicine, Dali University, Dali, Yunnan Province 671000 People’s Republic of China; 5 College of Veterinary Medicine, Jilin Agricultural University, ChangchunJilin Province, 130118 People’s Republic of China; 6 College of Veterinary Medicine, Qingdao Agricultural University, Qingdao, Shandong Province 266109 People’s Republic of China; 7 College of Animal Science and Veterinary Medicine, Heilongjiang Bayi Agricultural University, Heilongjiang Province163316, Daqing, People’s Republic of China

**Keywords:** *Toxoplasma gondii*, RNA sequencing, Long non-coding RNAs, Co-location, Liver, Stage-specific characteristics

## Abstract

**Background:**

*Toxoplasma gondii* is an intracellular protozoan parasite that can infect a wide range of warm-blooded animals, including humans. It poses significant health risks, particularly in immunocompromised individuals and during pregnancy, leading to severe disease manifestations. The liver, being a crucial organ involved in immune response and metabolic regulation, plays a critical role in the host's defense against *T. gondii* infection.

**Methods:**

In this study, we utilized RNA sequencing to investigate the expression profiles of long non-coding RNAs (lncRNAs) and messenger RNAs (mRNAs) in the liver of mice infected with *T. gondii*. By employing this method, we obtained a comprehensive overview of the alterations in gene expression occurring in the liver during infection.

**Results:**

By comparing the infected groups to the control groups, we identified numerous differentially expressed lncRNAs DElncRNAs and DEmRNAs at two stages of infection. Specifically, at the acute infection stage, we found 628 DElncRNAs, and 6346 DEmRNAs. At the chronic infection stage, we identified 385 DElncRNAs and 2513 DEmRNAs. Furthermore, we identified 1959 commonly expressed DEmRNAs, including *IL27*, *Nos2*, and *Cxcr2*, across two infection stages. Enrichment and co-location analyses revealed pathways linked to immune and inflammatory responses during *T. gondii* infection. Notably, through co-location analysis, our analysis revealed several DElncRNAs, including Gm29156, Gm29157, and Gm28644, which are potentially implicated in the progression of liver inflammation induced by *T. gondii*. Additionally, functional enrichment analysis disclosed stage-specific characteristics of liver inflammation and immune response, alongside changes in metabolic regulation and immunosuppression pathways.

**Conclusions:**

Our findings provide valuable insights into the expression patterns of lncRNAs and mRNAs in the liver at different stages of *T. gondii* infection. We identified potential regulatory factors and pathways implicated in liver inflammation, thereby enhancing our understanding of the molecular mechanisms underlying liver inflammation and immune responses during *T. gondii* infection. These findings could contribute to the development of targeted therapeutic strategies for liver inflammation in the context of *T. gondii* infection.

**Graphical Abstract:**

**Figure:**
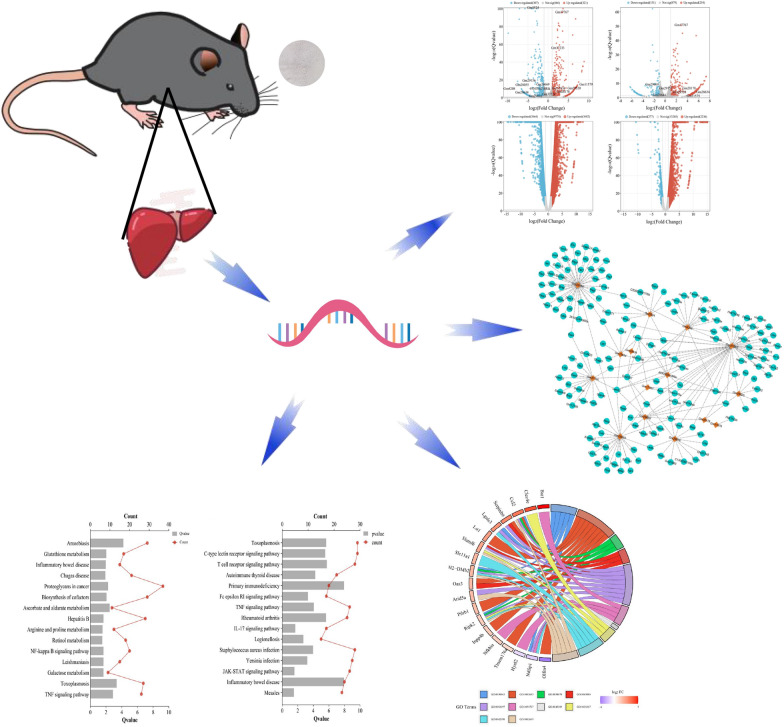

**Supplementary Information:**

The online version contains supplementary material available at 10.1186/s13071-023-06053-z.

## Background

Toxoplasmosis is a parasitic disease caused by the protozoan *Toxoplasma gondii*, with the ability to invade various mammals, including humans. Infection in humans is usually asymptomatic, but can be severe or even fatal in immunocompromised individuals or during pregnancy [1]. However, in rodents, particularly mice, toxoplasmosis can cause liver damage and other clinical manifestations [2]. Studies have shown that *T. gondii* infection can lead to hepatocyte necrosis, inflammation, and fibrosis in the liver, which can result in liver dysfunction [2]. The liver is an important organ for maintaining metabolic homeostasis and detoxification, and damage to the liver can have serious consequences for overall health. The mechanism of *T. gondii*-induced liver damage is not completely understood, but it is thought to involve a complex interplay between the parasite and the host immune response. The parasite is known to trigger the release of cytokines and chemokines, which can attract immune cells to the liver and cause inflammation [3].

Recent studies have suggested that both long non-coding RNAs (lncRNAs) and messenger RNAs (mRNAs) play important roles in the pathogenesis of *T. gondii* infection [4]. Several lncRNAs and mRNAs have been identified to be differentially expressed in the liver during the course of infection with *T. gondii* [2, 5]. For example, a study found that the mRNA for the pro-inflammatory cytokine *TNF-α* was significantly upregulated in *T. gondii*-infected livers [6]. *TNF-α* is known to promote liver inflammation and can contribute to liver damage [7]. However, a study has identified differentially expressed microRNA (miRNA) involved in pathways such as oxidative stress, apoptosis, and lipid metabolism in *T. gondii*-infected livers [8].

The available knowledge regarding the diverse signaling pathways and immune responses controlled by differentially expressed lncRNAs (DElncRNAs) and differentially expressed RNAs (DEmRNAs) during liver infection caused by *T. gondii* is limited. The identification of DElncRNAs and DEmRNAs during *T. gondii* infection in the liver and elucidation of their functions could provide new insights into the mechanisms underlying *T. gondii* pathogenesis in the liver and lead to the development of novel therapeutic strategies for toxoplasmosis-associated liver injury. Thus, we conducted RNA sequencing (RNA-Seq) analysis on liver samples obtained from mice infected with *T. gondii* at two stages post-infection. By analyzing both lncRNAs and mRNAs simultaneously, we were able to examine the correlation between their expression and the role of lncRNA/mRNA interactions in the pathogenesis of liver disease induced by *T. gondii* infection.

## Methods

### Ethics approval

The present study was approved by the Animal Ethics Committee of Qingdao Agricultural University.

### Construction of *Toxoplasma gondii* infection model and sample collection

*Toxoplasma gondii* (Prugniaud, PRU) cysts were acquired from the brains of mice that had undergone prolonged infection with the parasite. Then, 8–10-week-old specific-pathogen-free (SPF) female BALB/c mice were infected with 20 *T. gondii* cysts to construct the infection model. The mice were given unrestricted access to both food and water and housed in cages equipped with a self-contained ventilation arrangement. They were maintained on a 12-h light/dark cycle. The experiment consisted of three groups of mice: (1) a control group (*n* = 3), which were not infected with *T. gondii* and were used as a baseline for comparison; (2) an acute infection group (*n* = 3), which were monitored for signs of acute infection (11 days); and (3) a chronic infection group (*n* = 3), which was monitored for chronic infection over a period of 33 days. The mouse model was determined by amplification of the *T. gondii B1* gene. In this process, a direct polymerase chain reaction (PCR) approach was employed, utilizing a specific primer pair. This primer pair included a forward primer (5′-GGAACTGCATCCGTT-CATGAG-3′) and a corresponding reverse primer (5′-TCTTTAAAGCGTTCGTGGTC-3′). Subsequently, a semi-nested PCR was carried out. During this phase, the forward primer (5′-TGCATAGGTTGCAGTCACTG-3′) was used, while the reverse primer was preserved from the initial PCR round, following the methodology described in a prior study [9]. The timing of acute and chronic infection in the mouse model was determined based on the findings from a previous study [9]. At the designated time points following infection, mice from each group were euthanized, and their livers were dissected and rinsed with saline to remove blood from the liver surface. Subsequently, a small portion of the liver samples was immediately frozen in liquid nitrogen and stored until further RNA isolation.

### RNA isolation and sequencing

Total RNA was extracted from each liver sample using TRIzol reagent (Life Technologies, Carlsbad, CA, USA). The extracted RNA was assessed for degradation and contamination using 1% agarose gel electrophoresis. The RNA concentration was determined using the Qubit^®^ RNA Assay Kit on the Qubit^®^ 2.0 Fluorometer (Life Technologies, CA, USA), while the RNA integrity was evaluated using the RNA 6000 Nano assay kit on the Agilent 2100 Bioanalyzer system (Agilent Technologies, CA, USA). Only RNA samples with an RNA integrity number (RIN) ≥ 8 were used for sequencing analysis. Ribosomal RNA (rRNA) was removed by the Epicentre Ribo-Zero™ rRNA Removal Kit (Madison, WI, USA), and rRNA-free residue was cleaned up by ethanol precipitation.

For each lncRNA library, 3 μg of rRNA-depleted RNA was used for library construction with the NEBNext^®^ Ultra™ Directional RNA Library Prep Kit for Illumina^®^ (New England Biolabs, USA). First-strand complementary DNA (cDNA) was synthesized using M-MuLV Reverse Transcriptase (RNase H-) and random hexamer primers. The cDNA fragments, ranging from 150 to 200 base pairs (bp), were then purified using the AMPure XP system (Beckman Coulter, Beverly, MA, USA), followed by PCR amplification using Phusion High-Fidelity DNA polymerase. The resulting PCR products were purified using the AMPure XP system. The quality of the library was assessed using the Agilent 2100 Bioanalyzer system.

### Identification of transcripts

Initial fastq-formatted raw data was acquired and subjected to processing to extract clean data, involving the removal of adapters, poly-N sequences, and reads of low quality. Quality assessment of the clean reads was performed by calculating the guanine–cytosine (GC) content. The *Mus musculus* reference genome was used to build an index for alignment of the clean reads using bowtie2 v2.2.8 and HISAT2 v2.0.4. The mapped reads from each sample were assembled using StringTie (v1.3.1). Cuffdiff (v2.1.1) was utilized to calculate fragments per kilobase of transcript per million mapped reads (FPKM) for both lncRNAs and mRNAs of each sample. LncRNA transcripts were selected based on splicing transcripts with exons ≥ 2 and lengths > 200 bp.

### Differential expression analysis

Differential expression analysis was carried out at each infection stage using DESeq2 (1.14.1) [10]. Transcripts exhibiting a Q-value of < 0.05 and an absolute log_2_ fold change of ≥ 1 were considered as DElncRNAs and DEmRNAs, respectively. This approach was used to avoid any redundancies in the analysis.

### Co-location of DElncRNA and DEmRNA and functional analysis

To predict the potential targeted genes of DElncRNAs and understand their functions in the liver of mice infected with *T. gondii*, we utilized the co-location of DEmRNAs–DElncRNAs within a 100-kilobase (kb) upstream and downstream region. This approach takes into account the ability of lncRNAs to alter the expression of nearby genes. We utilized the visualization functions of Cytoscape software, which included “Import Network,” “Import Table,” and the adjustment of network node/edge properties, to construct the network. Following that, an annotation analysis of the DEmRNAs was carried out using the GOseq R package to explore Gene Ontology (GO) categories [11]. Some immune or inflammation-related GO terms were annotated. Significantly enriched terms were determined by a *Q*-value less than 0.05. Additionally, we employed the Kyoto Encyclopedia of Genes and Genomes (KEGG) pathway enrichment analysis to identify enriched signaling pathways and map the genes using KOBAS 3.0 [12].

### Validation of quantitative real-time PCR

To confirm the RNA-Seq results, randomly selected highly expressed DEmRNAs and DElncRNAs from each of the two infection time points were subjected to verification using quantitative real-time PCR (qRT-PCR). To synthesize the first-strand cDNA of mRNA and lncRNA, the PrimeScript™ RT Reagent Kit along with the gDNA Eraser genomic DNA removal kit (Takara, Tokyo, Japan) and the lnRcute lncRNA First-Strand cDNA synthesis kit (TianGen, Beijing, China) were employed, respectively. The qRT-PCR was conducted on a LightCycler 480 (Roche, Basel, Switzerland), with 40 cycles of amplification. The mRNA underwent initial denaturation at 95 °C for 10 min, followed by template denaturation during the PCR cycle at 95 °C for 30 s, and annealing at 60 °C for 1 min. The lncRNA amplification process consisted of an initial denaturation step at 95 °C for 3 min, template denaturation at 94 °C for 5 s, and annealing at 60 °C for 15 s in 40 cycles. The specificity of amplification was confirmed by melting curve analysis in each reaction. The reference gene used was *L13A*. All reactions were carried out three times, and the relative expression level was determined using the 2^−ΔΔCt^ method [13].

## Results

### Differential analysis of transcripts

**Fig. 1:**
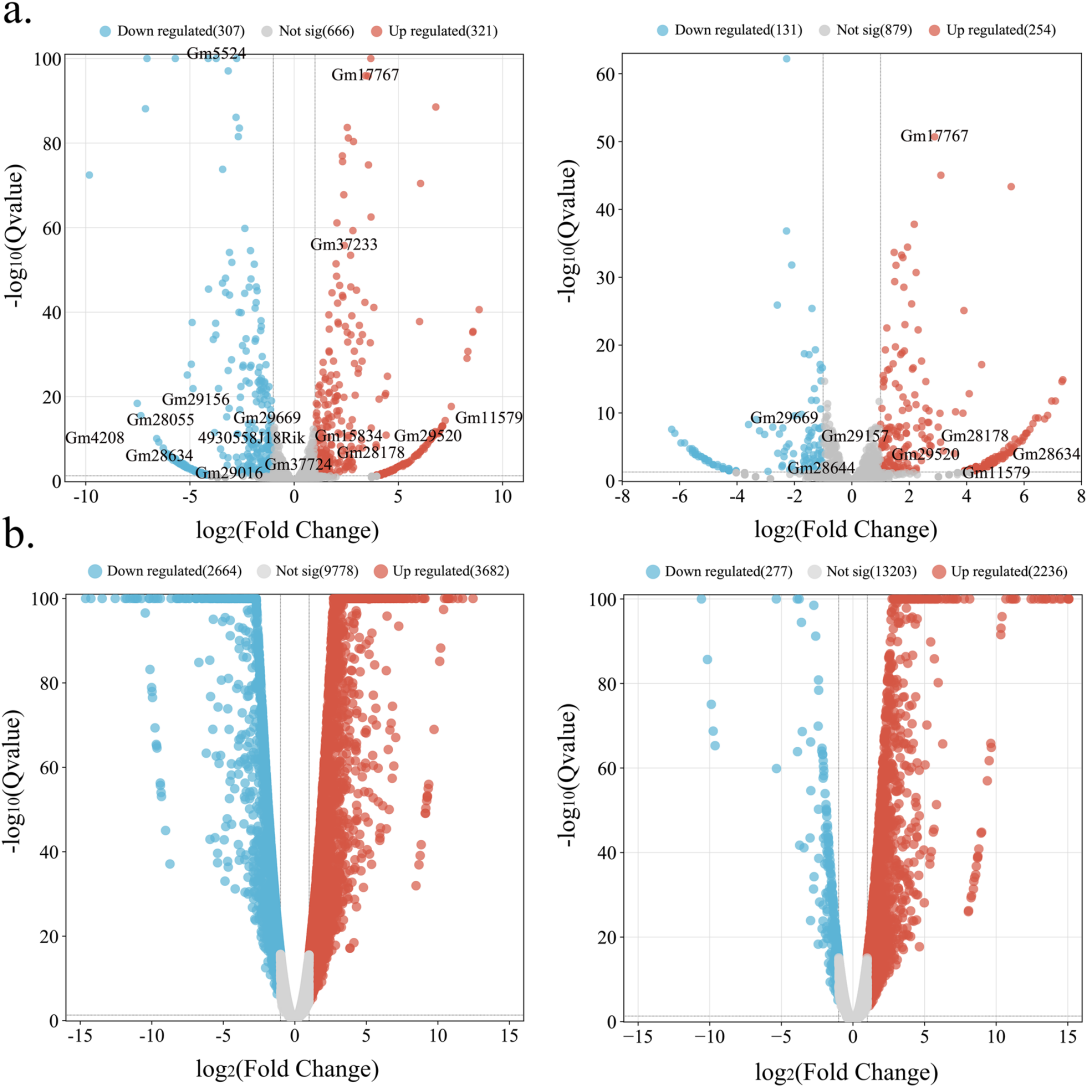
The volcano plots illustrate the differentially expressed lnRNAs (**a**) and differentially expressed mRNAs (**b**) during the acute and chronic stages of infection. The *x*-axis represents the log_2_ fold change of the differentially expressed transcripts, while the *y*-axis represents the corresponding −log_10_ Q-value. Upregulated transcripts are indicated in red, downregulated transcripts are indicated in blue, and transcripts that do not meet the significance threshold are displayed in gray

**Fig. 2:**
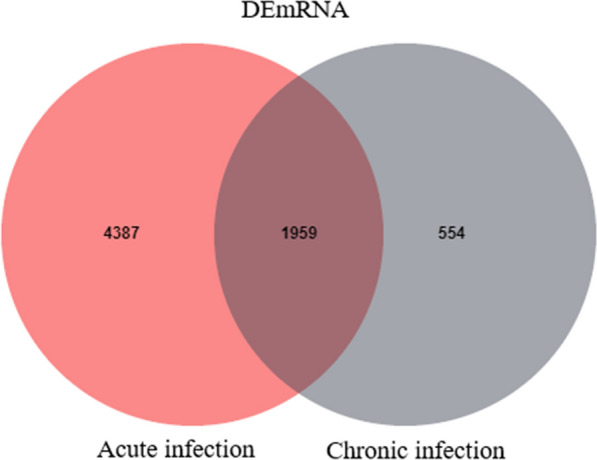
The Venn diagram depicts the overlapping and unique sets of the differentially expressed mRNAs during the acute and chronic stages of infection

After mapping to the *M. musculus* reference genome, the transcripts were divided into different subtypes. Compared to the control groups, a total of 628 DElncRNAs and 6346 DEmRNAs were identified at the acute infection stage; 385 DElncRNAs and 2513 DEmRNAs were identified at the chronic infection stage (Fig. 1 and Additional file 1: Table S1), of which 1959 mRNAs (e.g., *IL27*, *Nos2*, and *Cxcr2*) were commonly dysregulated between the acute and chronic infection groups (Fig. 2). Moreover, the *Cyp2c29* gene was downregulated at the acute infection stage.

### Co-location of DEmRNAs and DElncRNAs

**Fig. 3:**
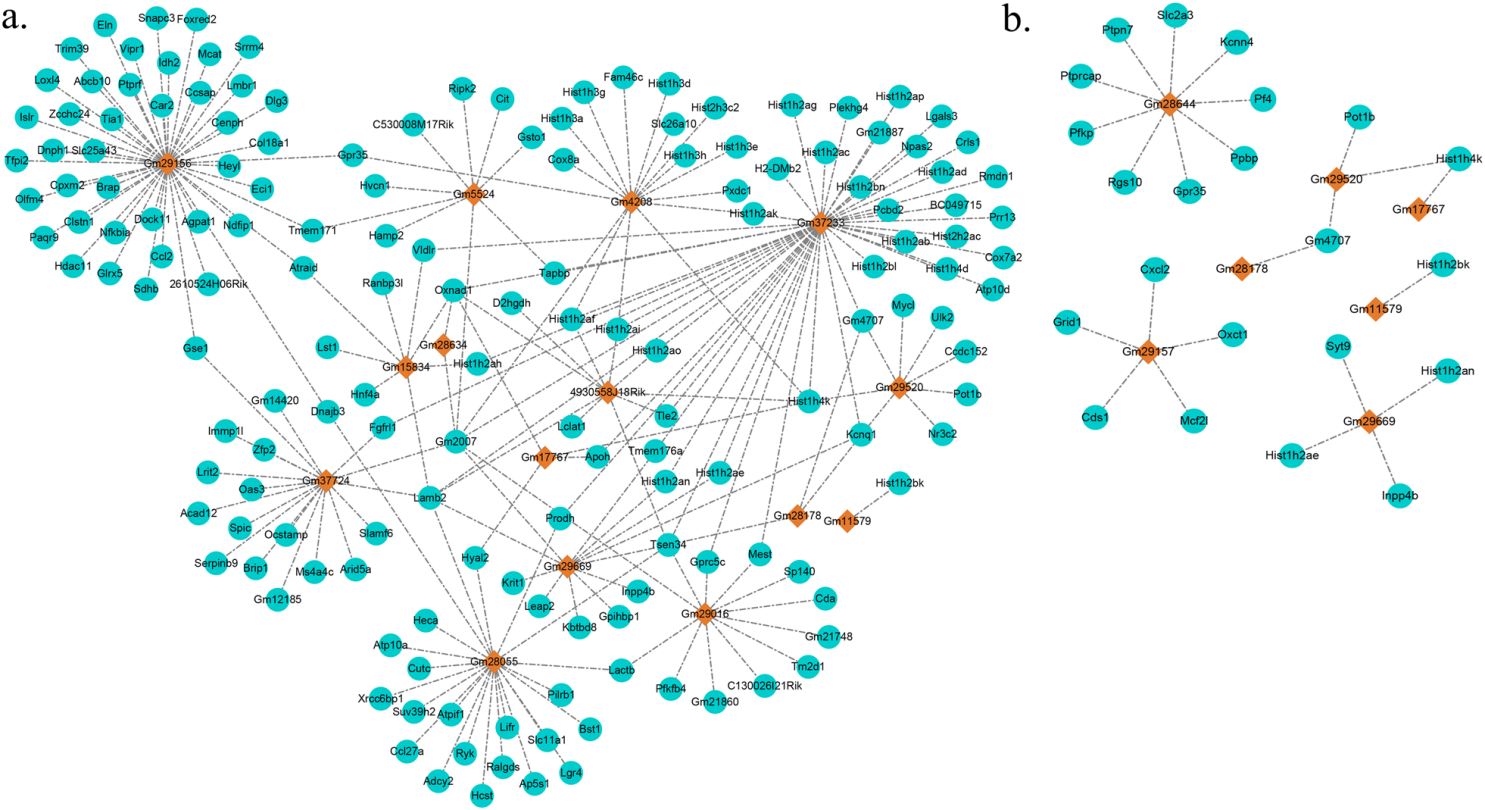
The co-location networks illustrate the interactions between differentially expressed lncRNAs (**a**) and differentially expressed mRNAs (**b**) in the liver during the acute and chronic stages of infection. In the network, the differentially expressed lncRNAs are represented by rhombus-shaped nodes, while the differentially expressed mRNAs are represented by circular nodes. The network visualizes the connections and relationships between these lncRNAs and mRNAs

At the acute infection stage, 214 DEmRNAs were located in the vicinity of 15 DElncRNAs (Fig. 3a and Additional file 2: Table S2). The multiple mRNAs were detected, and their regulation was found to be mediated by several lncRNAs. Among these DElncRNAs, the *Ccl2* and *Trim39* genes was located in the vicinity of Gm29156. *Slamf6* was located in the vicinity of Gm37724. In addition, the *Cox8a* gene was located in the vicinity of Gm4208. At the chronic infection stage, 24 DEmRNAs were located in the vicinity of seven DElncRNAs (Fig. 3b and Additional file 2: Table S2). The *Cxcl2* and *Oxct1* genes were located in the vicinity of the Gm29157. Also, the *Gpr35* gene was located in the vicinity of the Gm28644.

### The function of the DEmRNAs at two infection stages

**Fig. 4:**
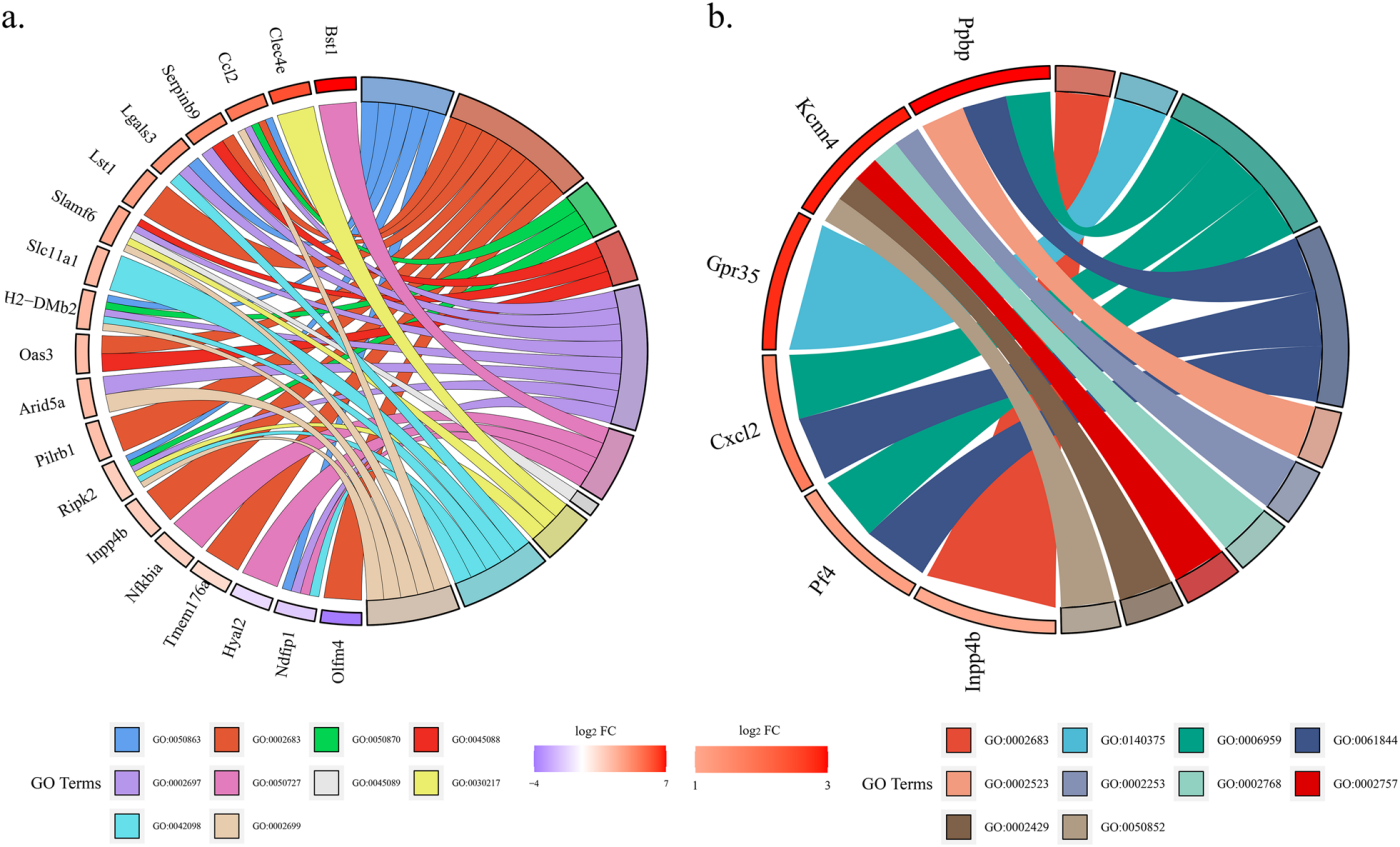
The chord diagram presents the gene ontology (GO) analysis results for the differentially represented mRNAs in the liver during the acute (**a**) and chronic (**b**) stages of infection. The diagram displays clustered genes along with their assigned immune- or inflammation-related GO terms, connected by ribbons. On the left side of the chord plot, the color gradient from blue to red represents the log_2_ fold change (log_2_ FC) of the genes. On the right side of the chord plot, various colors represent different GO terms associated with the genes

Function annotation revealed that some immune- or inflammation-related terms of mRNAs were observed at two infection stages. A total of 656 and 274 immune- or inflammation-related terms were observed at two infection stages (Additional file 3: Table S3). Among those GO terms, at the acute infection stage, some targeted DEmRNAs (e.g., *Ccl2*, *Lgals3*, *H2-DMb2*, *Ripk2*, and *Ndfip1*) were enriched in the “regulation of T cell activation” term (GO:0050863). The targeted DEmRNAs *Serpinb9*, *Slamf6*, and *Oas3* were significantly enriched in “regulation of innate immune response” (GO:0045088). In addition, targeted DEmRNAs such as *Bst1*, *Nfkbia*, *Hyal2*, and *Ndfip1* were enriched in “regulation of inflammatory response” (GO:0050727) (Fig. 4a). At the chronic infection stage, only a few targeted DEmRNAs were significantly enriched in the immune- or inflammation-related terms (Fig. 4b). The DEmRNAs *Ppbp*, *Cxcl2*, and *Pf4* were significantly enriched in “antimicrobial humoral immune response mediated by antimicrobial peptide” (GO:0061844), and “humoral immune response” (GO:0006959).

**Fig. 5:**
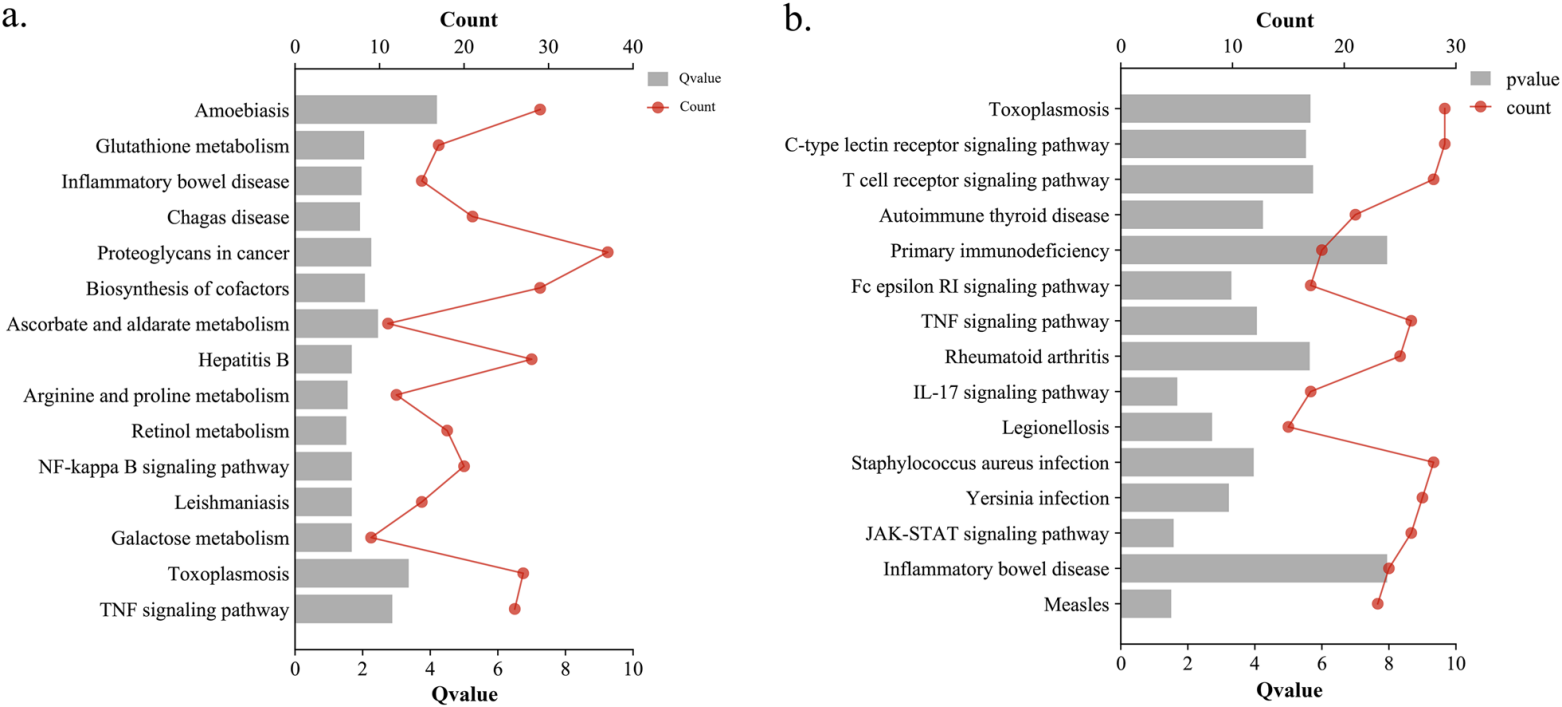
A dual *x*-axis bar chart showing KEGG pathway enrichment results for the differentially expressed (DE)mRNAs. The enriched pathways during the acute stage of infection (**a**) and the chronic stages of infection (**b**) are listed. The bottom *x*-axis is labeled with Q-values, the *y*-axis displays the names of KEGG pathways, and the top *x*-axis represents the counts of genes associated with each pathway

 For functional annotation of KEGG pathway enrichment, in the acute infection stage, we observed significant enrichment of upregulated mRNAs (*Gpx2* and *Gpx3*) in the glutathione metabolism pathway. Additionally, upregulated mRNAs *Ugt1a7c* and *Ugt1a8* were notably enriched in the ascorbate and aldarate metabolism pathway. Furthermore, targeted mRNAs *Arg1*, *Arg2*, *Ckb*, and *Nos2* exhibited upregulation and enrichment in the arginine and proline metabolism pathway, while targeted mRNAs *G6pc*, *Hk1*, *Hk3*, and *Pfkp* showed similar patterns in the galactose metabolism pathway. These pathways are closely related to liver metabolism. Moreover, targeted mRNAs *Ifng* and *Nos2* displayed upregulation and enrichment in several parasite infection pathways, including the amoebiasis, Chagas disease, leishmaniasis, and toxoplasmosis pathways (Fig. 5a and Additional file 1: Table S1). In the chronic infection stage, specific targeted mRNAs *Cd40* and *Cd40lg* were upregulated and significantly enriched in pathways associated with immunosuppression, including autoimmune thyroid disease and primary immunodeficiency pathways. Additionally, other immunosuppression-related pathways, such as rheumatoid arthritis, IL-17 signaling pathway, inflammatory bowel disease, and measles, were observed. Furthermore, the targeted mRNA *Pik3cd* displayed upregulation and enrichment in pathways associated with inflammation, including the Fc epsilon RI signaling pathway, TNF signaling pathway, and JAK-STAT signaling pathway, all of which were significantly enriched (Fig. 5b and Additional file 1: Table S1).

### Quantitative real-time PCR verification

DElncRNAs and DEmRNAs exhibiting higher expression levels were randomly selected for qRT-PCR verification (Additional file 4: Fig. S1), including *Ubd*, *Chil3*, *Ly6i*, *Saa1*, GM29156, GM4208, and GM29157. Although the expression levels of the DElncRNAs and mRNAs obtained by qRT-PCR were slightly lower than those obtained by RNA-Seq, most of the expression trends obtained by both methods were consistent.

## Discussion

*Toxoplasma gondii* is an intracellular parasite that can infect a wide range of hosts, including humans and mice. In mice, liver infection with *T. gondii* is a well-established model for studying the host−parasite interaction and the underlying molecular mechanisms involved. LncRNAs and mRNAs are two important classes of RNA molecules that play essential roles in gene regulation and cellular processes. The present study aimed to identify specific lncRNAs and mRNAs that are differentially expressed upon infection, which can provide insights into the host response to the parasite and potential therapeutic targets.

In the current study, we observed that the expression of the *IL27* gene was upregulated 20.13-fold and 7.68-fold at the acute and chronic infection stage, respectively. *IL-27* is initially characterized as a cytokine with pro-inflammatory properties that can induce the differentiation of TH1 cells [14]. Previous research suggested that *IL-27* could initiate immune responses to protect against hepatic injury [15]. In contrast, an additional study demonstrated a positive correlation between high levels of *IL-27* and Th17 cells, which may serve as an indicator of liver injury in patients infected with hepatitis B virus (HBV) [16]. These findings suggest that *IL-27* might play a role in liver injury induced by *T. gondii*. The *NOS2* gene encodes inducible nitric oxide synthase (iNOS), which is an enzyme that catalyzes the production of nitric oxide (NO) in response to various stimuli. An in vitro study has demonstrated the significance of inducible *NOS2* in the production of NO, which plays a crucial role in inhibiting the proliferation of *T. gondii* tachyzoites in macrophages [17]. Another study has shown that the activities mediated by *NOS2* in innate immune cells are crucial for suppressing the formation of cysts in the brain [18]. The expression of the *NOS2* gene was upregulated 205.38-fold at the acute infection stage and decreased to 23.79-fold at the chronic infection stage, which showed that the liver could produce *NOS2* to resist *T. gondii* at the acute infection stage. Moreover, the *Cxcr2* gene plays a crucial role in the initial recruitment of neutrophils, contributing significantly to the body's defense against *Toxoplasma*. This chemokine receptor, along with its ligands, plays a protective role in resistance against this pathogen during the early stages of infection [19]. In this study, the *Cxcr2* gene was upregulated 7.54-fold at acute infection stages. This finding suggests that the liver can express the *Cxcr2* gene to resist *T. gondii* infection. In addition, transcriptional levels of *Cyp2c29* were observed to be downregulated in a mouse model of nonalcoholic steatohepatitis [20]. A previous study provided evidence indicating that the suppression of the inflammatory response by *Cyp2c29* may contribute to a reduction in liver injury [21]. In this study, *Cyp2c29* was downregulated by 34.23-fold at the acute infection stage and returned to normal levels at the chronic infection (fold change < 1). These findings further support the notion that *T. gondii* induces liver inflammation at the acute infection stage.

As lncRNAs are able to modulate the expression of neighboring genes, potential targeted genes for the DElncRNAs were forecasted based on the co-location with mRNAs−DElncRNAs situated within a range of 100 kb upstream and downstream. The *Ccl2* gene was upregulated 25.9-fold by Gm29156 at the acute infection stage. The *Ccl2* is the strongest chemoattractant involved in macrophage recruitment and a powerful initiator of inflammation [22]. *Ccl2*/CCR2 signaling is primarily recognized for its pivotal function in the modulation of macrophage recruitment and polarization in inflammatory processes [23]. The significance of monocytes is indicated by the discovery that mice deficient in CCR2, the receptor for *Ccl2*, exhibit heightened susceptibility to *T. gondii* infection [24]. These observations suggested that Gm29156 may modulate the expression of the *Ccl2* gene, contributing to the development of liver inflammation induced by *T. gondii*. Furthermore, macrophage inflammatory protein 2 (MIP-2) is a member of the CXC chemokine family and is alternatively referred to as chemokine CXC ligand 2 (*Cxcl2*) [25]. The *Cxcl2* plays a crucial role in inflammation by attracting and activating immune cells, particularly neutrophils, to sites of injury or infection [26, 27]. It aids in the recruitment of polymorphonuclear neutrophils (PMNs) to areas of injury or infection, thus regulating immune and inflammatory responses. It acts as a mediator of liver inflammation at high concentrations, contributing to the inflammatory cascade and exacerbating liver damage. However, at lower concentrations, MIP-2/*Cxcl2* switches its role and promotes liver regeneration by facilitating the recruitment and activation of regenerative cells [25]. This dual role highlights the intricate balance between inflammatory processes and tissue repair mechanisms in liver diseases, emphasizing the multifaceted nature of MIP-2/*Cxcl2* in liver pathophysiology. In the chronic infection stage, the Gm29157 exhibits the ability to enhance the expression of *Cxcl2*, albeit to a modest extent (only 3.53-fold). This upregulation of *Cxcl2* expression plays a crucial role in promoting liver regeneration, particularly when present at lower concentrations. The involvement of Gm29157 and its impact on *Cxcl2* highlight the intricate regulatory mechanisms underlying liver regeneration during *T. gondii* chronic infection, further emphasizing the multifaceted nature of these molecular interactions in liver pathophysiology. It has been documented that *Slamf6* can stimulate the activation of natural killer cells, leading to the promotion of cytotoxicity and modulation of IFN-γ production [28]. Furthermore, *Slamf6* can facilitate Th17 differentiation. Additionally, it can promote the interaction between colonic innate immune cells and Gram-negative bacteria, consequently diminishing mucosal protection and amplifying inflammation, ultimately leading to lethal colitis in mice [29, 30]. At the acute infection stage, *Slamf6* exhibited a notable 3.57-fold upregulation, a regulation attributed to Gm37724. These findings shed light on the role of Gm37724 in promoting *Slamf6* expression, thereby contributing to liver inflammation during *T. gondii* infection. *Gpr35* has been suggested as a potential risk factor associated with chronic inflammatory conditions of the gastrointestinal tract, such as inflammatory bowel disease (IBD) and ulcerative colitis [31]. *Gpr35* has been associated with inflammation, particularly in studies demonstrating that the administration of *Gpr35* agonists can reduce inflammatory processes, implying that *Gpr35* possesses the ability to modulate inflammatory conditions [32]. The upregulation of *Gpr35* by 6.13-fold, induced by Gm28644 during the chronic infection stage, further strengthens the connection between *Gpr35* and inflammatory conditions. This finding suggests that the modulation of *Gpr35* expression by Gm28644 may play a role in regulating inflammatory processes associated with *T. gondii* chronic infection. The precise mechanisms by which *Gpr35* and Gm28644 interact to influence inflammatory responses warrant further investigation, highlighting the potential of targeting this molecular pathway for therapeutic interventions in chronic inflammatory disorders.

The GO function annotation analysis uncovered supplementary predictions indicating that the DEmRNA plays a crucial role in the liver during *T. gondii* infection. To investigate potential regulatory factors and pathways involved in liver inflammation and immunity during *T. gondii* infection, our focus was on specific mRNA terms related to immunity and inflammation that were observed at two infection stages. In the acute infection stage, a greater number of DEmRNAs were observed to be enriched in Gene Ontology (GO) categories associated with immunity and inflammation. However, in the chronic infection phase, a lower number of DEmRNAs were found to be enriched in these GO entries, indicating a potential shift or decrease in the immune and inflammatory response over time. Furthermore, during the acute infection stage, the genes *Arg1*, *Arg2*, *Ckb*, and *Nos2* displayed upregulation and were found to be enriched in the arginine and proline metabolism pathway. Arginine metabolism in the liver also serves as a modulator of the immune response. Arginine serves as a precursor for the synthesis of nitric oxide (NO), which possesses immune−regulatory functions. The liver has the capacity to generate NO to combat infection and modulate immune responses [33]. This suggests that *Arg1*, *Arg2*, *Ckb*, and *Nos2* play a pivotal role in activating liver metabolic processes that contribute to resistance against *T. gondii* infection. Interestingly, in the chronic infection stage, the presence of certain immunosuppression pathways, including autoimmune thyroid disease, primary immunodeficiency, rheumatoid arthritis, IL-17 signaling pathway, inflammatory bowel disease, and measles, was observed. This indicates that during prolonged *T. gondii* infection, there may be mechanisms at play that suppress the immune response. Understanding these immunosuppression pathways can provide insights into the complex interplay between the host immune system and *T. gondii* during chronic infection.

## Conclusions

In the present study, we conducted a thorough examination of lncRNA and mRNA expression profiles in the livers of mice infected with *T. gondii* at two different stages of infection. Through co-location analysis, we identified several DElncRNAs that potentially contribute to the development of liver inflammation induced by *T. gondii*. Furthermore, functional enrichment analysis revealed that the liver inflammation and immune response triggered by *T. gondii* infection were accompanied by alterations in metabolic regulation and immunosuppression pathways. These findings shed light on the intricate molecular mechanisms underlying the liver's response to *T. gondii* infection and provide insights into the dynamic changes that occur during different stages of the infection process.

## Supplementary Information

**Additional file 1: Table S1.** The differentially expressed lncRNAs and mRNAs in livers of mice during the acute and chronic stages of infection.

**Additional file 2: Table S2.** The co-location of the differentially expressed lncRNAs and mRNAs in the liver of mice during the acute and chronic stages of infection.

**Additional file 3: Table S3.** The immune- or inflammation-related GO terms of the differentially expressed mRNAs in the liver of mice during the acute and chronic stages of infection.

**Additional file 4: Figure S1.** The quantitative real-time PCR (qRT-PCR) verification of the differentially expressed mRNAs and lncRNAs at the acute and chronic stages of infection.

## Data Availability

The RNA-Seq raw data of the repository/repositories and accession number(s) can be found below: https://www.ncbi. nlm.nih.gov/bioproject/PRJNA876783, and PRJNA876593.
