# Correction: Transcriptomic profiling of long non-coding RNAs and messenger RNAs in the liver of mice during *Toxoplasma gondii* infection

**DOI:** 10.1186/s13071-024-06516-x

**Published:** 2024-10-08

**Authors:** Yang Zou, Xing Yang, Chao Chen, He Ma, Hong-Wei Cao, Jing Jiang, Xin-Yu Wei, Xiao-Xuan Zhang

**Affiliations:** 1 School of Pharmacy, Yancheng Teachers University, Yancheng, 224002 Jiangsu Province People’s Republic of China; 2 School of Life Sciences, Baicheng Normal University, Baicheng, 137000 Jilin Province People’s Republic of China; 3 State Key Laboratory of Veterinary Etiological Biology, Key Laboratory of Veterinary Parasitology of Gansu Province, Lanzhou Veterinary Research Institute, Chinese Academy of Agricultural Sciences, Lanzhou, 730046 Gansu Province People’s Republic of China; 4 Department of Medical Microbiology and Immunology, School of Basic Medicine, Dali University, Dali, 671000 Yunnan Province People’s Republic of China; 5 College of Veterinary Medicine, Jilin Agricultural University, Changchun, 130118 Jilin Province People’s Republic of China; 6 College of Veterinary Medicine, Qingdao Agricultural University, Qingdao, 266109 Shandong Province People’s Republic of China; 7 College of Animal Science and Veterinary Medicine, Heilongjiang Bayi Agricultural University, Daqing, 163316 Heilongjiang Province People’s Republic of China

**Correction: Parasites & Vectors (2023) 17:20** 10.1186/s13071-023-06053-z

Following publication of the original article [1], it was brought to the attention of the journal that there was an error in Additional file 1. Namely, ‘Sheet 3’ had been erroneously replaced by a duplicate of ‘Sheet 4’. The file has since been corrected in the published article (that is, ‘Sheet 3’ has been updated with the correct values). The authors thank you for reading this erratum and apologize for any inconvenience caused.
