# Supplementary figures and images for: Transcriptomic profiling of long non-coding RNAs and messenger RNAs in the liver of mice during Toxoplasma gondii infection
Source: Parasit Vectors. 2024 Jan 16;17:20. doi: 10.1186/s13071-023-06053-z (PMC10792800; doi:10.1186/s13071-023-06053-z)

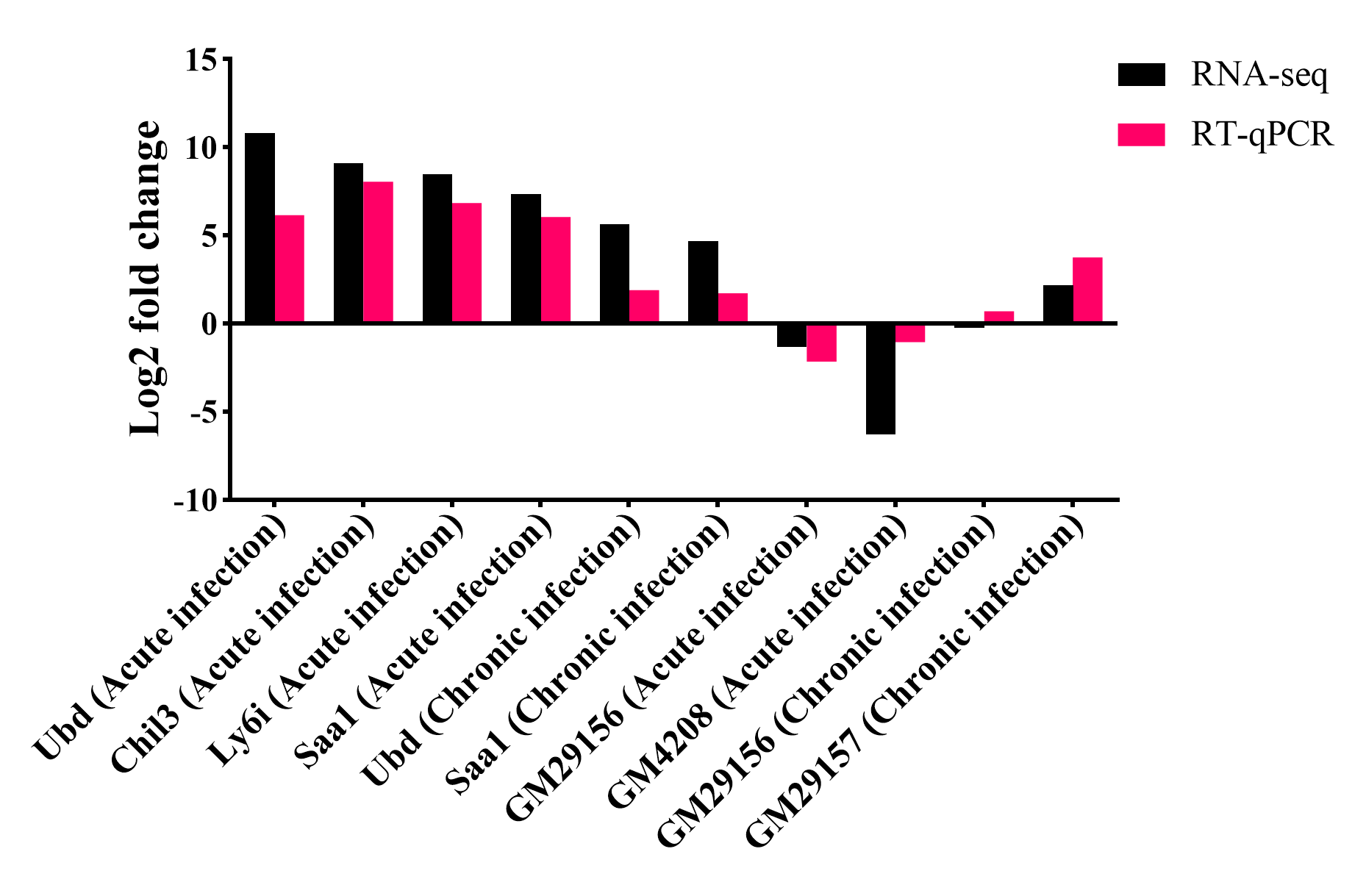

Supplement: Supplementary file 4 — Additional file 4: Figure S1. The quantitative real-time PCR (qRT-PCR) verification of the differentially expressed mRNAs and lncRNAs at the acute and chronic stages of infection. [file 13071_2023_6053_MOESM4_ESM.tif]
